# A tomato MADS-box protein, SlCMB1, regulates ethylene biosynthesis and carotenoid accumulation during fruit ripening

**DOI:** 10.1038/s41598-018-21672-8

**Published:** 2018-02-21

**Authors:** Jianling Zhang, Zongli Hu, Qiyuan Yao, Xuhu Guo, Vanluc Nguyen, Fenfen Li, Guoping Chen

**Affiliations:** Laboratory of molecular biology of tomato, Bioengineering College, Chongqing University, Chongqing, People’s Republic of China

## Abstract

The MADS-box transcription factors play essential roles in many physiological and biochemical processes of plants, especially in fruit ripening. Here, a tomato MADS-box gene, *SlCMB1*, was isolated. *SlCMB1* expression declined with the fruit ripening from immature green to B + 7 (7 days after Breaker) fruits in the wild type (WT) and was lower in *Nr* and *rin* mutants fruits. Tomato plants with reduced *SlCMB1* mRNA displayed delayed fruit ripening, reduced ethylene production and carotenoid accumulation. The ethylene production in *SlCMB1*-RNAi fruits decreased by approximately 50% as compared to WT. The transcripts of ethylene biosynthesis genes (*ACS2*, *ACS4*, *ACO1* and *ACO3*), ethylene-responsive genes (*E4*, *E8* and *ERF1*) and fruit ripening-related genes (*RIN*, *TAGL1*, *FUL1*, *FUL2*, *LoxC* and *PE*) were inhibited in *SlCMB1*-RNAi fruits. The carotenoid accumulation was decreased and two carotenoid synthesis-related genes (*PSY1* and *PDS*) were down-regulated while three lycopene cyclase genes (*CYCB*, *LCYB* and *LCYE*) were up-regulated in transgenic fruits. Furthermore, yeast two-hybrid assay showed that SlCMB1 could interact with SlMADS-RIN, SlMADS1, SlAP2a and TAGL1, respectively. Collectively, these results indicate that SlCMB1 is a new component to the current model of regulatory network that regulates ethylene biosynthesis and carotenoid accumulation during fruit ripening.

## Introduction

Fruit ripening has always been the focus of scientific research, which is mainly due to not only the uniqueness of this biological process but also the important role that fruits provide nutrition for animal and human^1,2^. The biological study of fruit ripening have resulted in significant gains in knowledge over recent years. Fruit ripening is a complex physiological and biochemical process with many metabolic changes (e.g. color, flavor, texture, aroma and nutrition) which are regulated by genetic regulators, external signals and endogenous hormones^3,4^. Typically, two classes of fruits have been identified as the non-climacteric fruits (e.g. pepper and strawberry) and the climacteric fruits (eg. pear and tomato). In climacteric fruits, ethylene biosynthesis and respiration increased remarkably at the beginning of fruit ripening and the burst in ethylene production is necessary for normal ripening of fruits, while in non-climacteric fruits, these changes are not observed^3^.

Ethylene is an important plant endogenous hormone which plays significant regulatory roles in the growth and development of plants, such as flavor generation, fruit ripening, leaf senescence, and other programmed signals of senescence and defense^5–9^. It is well known that ethylene is a key regulatory factor at the beginning of ripening and is necessary for the process of fruit ripening^10,11^. To date, ACS (1-AMINOCYCLOPROPANE-1-CARBOXYLATE SYNTHASE) and ACO (1-AMINOCYCLOPROPANE-1-CARBOXYLATE OXIDASE) are identified as two kinds of key enzymes in ethylene biosynthesis^12–14^. Alexander *et al*. (2002) have reported that fruit ripening and ethylene biosynthesis are markedly repressed when the expression of *SlACS2* was suppressed significantly in *SlACS2*-RNAi lines. Moreover, some studies have shown that exogenous ethylene can evidently induce the accumulation of *SlACS2* transcripts^15–17^. It has been revealed that the transcripts of *SlACO1* and *SlACO3* are markedly accumulated when tomato fruit ripening is triggered^15,18^. In addition, previous studies manifest that *SlACO1* positively regulates tomato fruit ripening^2,19^, when the expression of *SlACO1* was suppressed in transgenic tomato fruits, the biosynthesis of endogenous ethylene was decreased and the storage ability of tomato fruits increased^20^. In addition to ethylene synthesis, ethylene response and perception are also essential to the ripening of fruits. *E4* and *E8*, which are induced by ethylene, are generally considered as two classical genes of ethylene perception and response^21^. Many studies have shown that the accumulation of *E4* transcripts is significantly induced by exogenous ethylene^22,23^. Also the expression of *E4* is inhibited when the biosynthesis of ethylene is suppressed^24^. *E8*, which takes part in the feedback regulation of ethylene biosynthesis, is a fruit ripening-specific expression gene and is activated when fruit ripening is triggered^25^. After being characterized, the *E8* promoter is widely used as a fruit-specific promoter to drive the transcripts of exogenous genes to study their function in transgenic tomato^26,27^.

Tomato is generally used as an excellent model plant for fruit ripening study, not only because of several desirable attributes, such as short life cycle, small genome size, efficient stable transformation, high-density genetic maps and the completion of tomato genome sequence^28–30^, but also the existence of lots of well-characterized ripening tomato mutants, such as *Green ripe* (*Gr*), *never ripe* (*Nr*), *color nonripening* (*cnr*) and *ripening inhibitor* (*rin*), have been found and studied^31–35^. These superiority and mutants of tomato help us to reveal the mechanism of fruit ripening^36^. The *rin* mutant tomato plants exhibit enlarged sepals, an altered inflorescence architecture and inhibited fruit ripening due to the absence of two functional MADS-box genes, *SlMADS-MC* and *SlMADS-RIN*. Loss one of these MADS-box gene, the *SlMADS-RIN*, results in failure of fruit ripening, whereas loss of the other, *SlMADS-MC*, the phenotypes of enlarged sepals and altered inflorescence architecture are observed^32^.

The MADS-box genes, encoding DNA-binding transcription factors, which have been characterized from the genome of plants, animals and fungi, play significant roles in numerous biological processes^37^. In tomato, at least 36 functional MADS-box transcriptional factors have been characterized and analyzed^38^. Among tomato MADS-box genes, many of these, such as *TAGL1* (*TOMATO AGAMOUS LIKE1*), *FUL1*(*FRUITFULL1*), *FUL2*, *SlFYFL* and *SlMADS1*, have been studied and identified to be involved in fruits development or/and ripening. Overexpression of the *TAGL1* gene leads to enhanced lycopene in tomato fruits and the transition of sepals into succulent fruit organs, but when the transcripts of *TAGL1* are suppressed using RNAi approach, the transgenic tomato fruits display the phenotype of reduced pericarp thickness, altered starch accumulation and ripening inhibition^39–41^. Recently, a new study reported that *TAGL1* inhibits cuticle development and lignin biosynthesis^42^. Tomato *FUL1* and *FUL2* genes are the homolog of the *Arabidopsis FRUITFULL* (*FUL*) gene, Shima *et al*. have reported that there is functional redundancy between FUL1 and FUL2 and these two proteins could form heterodimers with RIN to regulate fruit ripening through regulating the expression of ripening-related genes^43^. Moreover, tomato FRUITFULL (FUL1 and FUL2) homologs and TAGL1 also are able to form high order complexes with RIN to regulate fruit ripening^44^. *SlFYFL* and *SlMADS1* are two MADS-box genes that participate in fruit ripening, overexpression of *SlFYFL* leads to the phenotypes of inhibited fruit ripening^45^ and suppression of *SlMADS1* results in ripening inhibition^46^.

The fruit ripening regulatory network requires a number of regulators that regulate each other or/and the expression of other ripening-related genes to successfully complete the fruit ripening process. Although many regulators of MADS-box family have been reported to participate in tomato fruit ripening, there are still so many ripening-related MADS-box genes need to be investigated. This will contribute to enriching the fruit ripening regulatory network and to further revealing the mechanism of fruit ripening regulation. Recently, a new study by Soyk, S. *et al*. showed that mutation of the SEP MADS-box gene, *J2* (accession no. Solyc04g005320) results in longer inflorescence in tomato (*S*. *pimpinellifolium*)^47,48^. The result of sequence alignment showed this *J2* gene which has been named as *SlCMB1* (accession no. XM_004237013) by the NCBI database is the investigated gene in our study. Interestingly, this gene was also found to be involved in tomato fruit ripening in addition to its roles in the architecture development in our study. To comprehensively investigate the diversified functions of MADS-box genes, *SlCMB1* was isolated from the wild type tomato (*Solanum lycopersicon* Mill. cv. Ailsa Craig). RNAi repression of *SlCMB1* was carried out to study the exact role of *SlCMB1* in fruit ripening of tomato, and our results showed that suppression of *SlCMB1* results in inhibited ethylene biosynthesis and reduced carotenoid accumulation during tomato fruit ripening. In addition, we analyzed the *SlCMB1*-RNAi lines at molecular and physiology levels. This paper enhanced our insights into the roles of *SlCMB1* playing in multiple biological processes.

## Results

### Molecular characterization of *SlCMB1*

**Figure 1:**
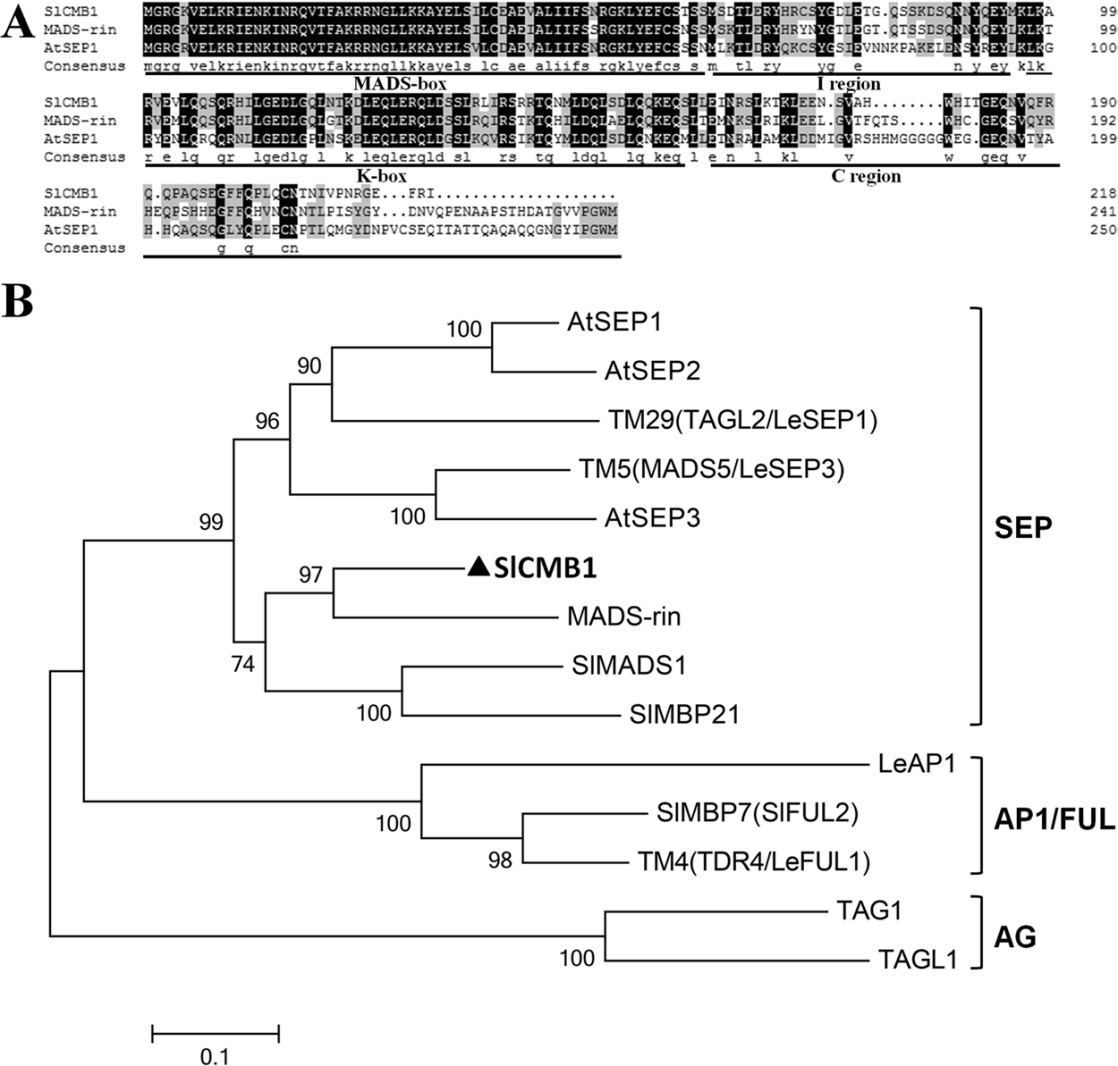
Multiple sequence alignment and phylogenetic analysis of SlCMB1 with other MADS-box proteins. (**A**) Multiple sequence alignment of SlCMB1 with two other MADS-box proteins. The black part are the identical amino acids, and the gray are the similar amino acids. The MADS-box, K-box, I region, and C region are identified. (**B**) Phylogenetic analysis of the SlCMB1 with other known MADS-box proteins. SlCMB1 is in bold. Accession numbers of these proteins are listed as follows: AtSEP1 (AED92207.1), AtSEP2 (AEE73791.1), AtSEP3 (AEE30503.1), MADS-RIN (AF448522), SlMBP21 (NP_001275579), SlMADS1 (NP_001234380), LeAP1 (AAP83378.1/AY306154), TM5 (MADS5/LeSEP3) (NP_001234384/AY306153), TM29 (TAGL2/LeSEP1) (NP_001233911/AY306152), LeFUL1 (AY098732.1), LeFUL2 (NM_001307938.1), TAG1 (L26295.1), TAGL1 (AY098735.2).

Based on the bioinformatics analysis of tomato MADS-box transcription factor family in our laboratory and the sequence on the NCBI (National Center for Biotechnology Information) web site, a tomato MADS-box gene was cloned from the wild type tomato (*Solanum lycopersicon* Mill. cv. Ailsa Craig). We named this gene as *SlCMB1* (accession number: XM_004237013) following the existing name at the NCBI database. The nucleotide sequence analyses indicated that *SlCMB1* contained a 717-bp ORF encoding a protein with 238 amino acids and this protein had an estimated molecular mass of 27.5 kDa (pI 8.62). Multiple alignment result showed that SlCMB1 had the typical MADS-box domains (i.e. the MADS domain, the K domain and the I domain) and the C-terminal region of SlCMB1 had significant difference to other known MADS-box proteins (Fig. 1A)^38,49^. In addition, phylogenetic analysis displayed that SlCMB1 belongs to the SEPALLATA (SEP) clade and showed higher similarity with SlMADS-RIN compared with other functional MADS-box proteins (Fig. 1B). Moreover, the result of promoter analysis showed that an 8 bp cis-regulatory element, ERE motif (ATTTCAAA) (Supplementary Fig. S1), which is an ethylene-responsive element was found at position −1844 in the promoter region of *SlCMB1* gene, indicating that *SlCMB1* gene might play an essential regulatory role in the process of fruit ripening.

### Expression pattern analysis of *SlCMB1*

**Figure 2:**
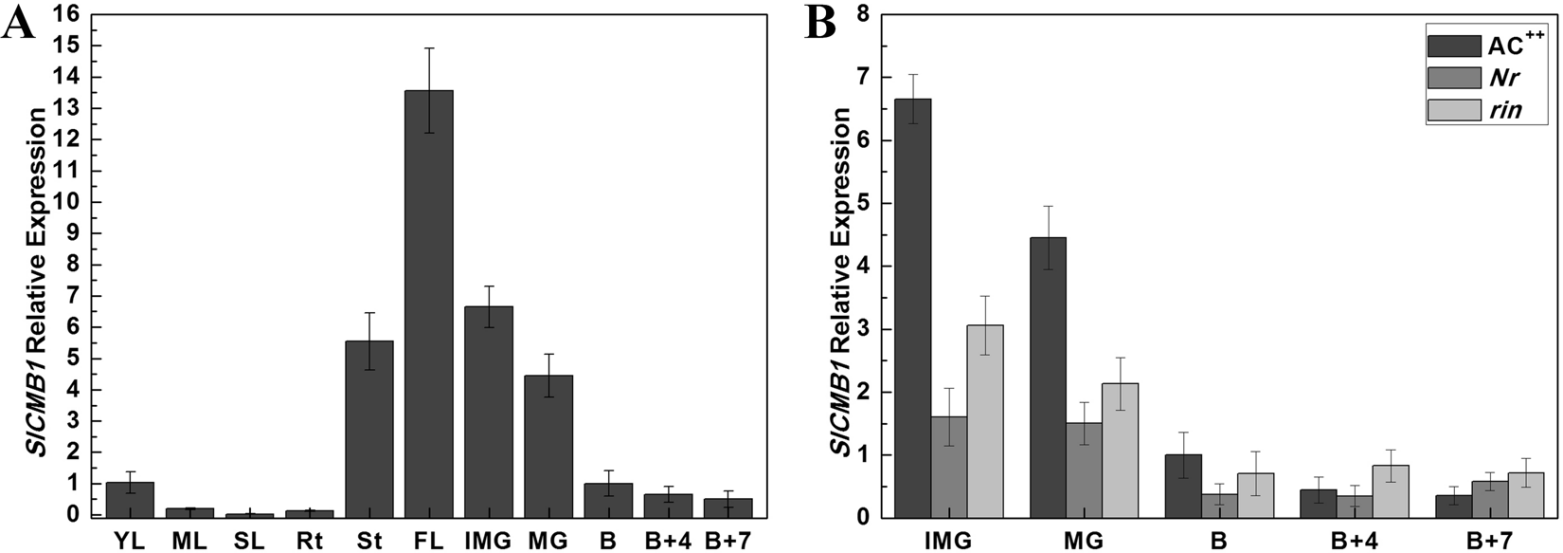
Expression patterns of *SlCMB1* in different tissues of AC^++^ (Mill. cv. Ailsa Craig) and ripening-related mutant fruits. (**A**) The expression of *SlCMB1* in YL, young leaves; ML, mature leaves; SL, senescent leaves; Rt, roots; St, stems; FL, flowers at anthesis; IMG, immature green fruits; MG, mature green fruits; B, breaker fruits; B + 4, fruits 4 days after breaker; B + 7, 7 days after breaker fruits in AC^++^. (**B**) Expression of *SlCMB1* in different stages of AC^++^, *Nr*, and *rin* fruits. The *SlCMB1* relative expression in B stage fruits of AC^++^ was set the control sample. Error bars indicate SE.

The tissue specificity of gene expression may be correlated with specific biological functions. Thus quantitative real-time PCR (qRT-PCR) was performed to analyze the transcripts of *SlCMB1* in different tissues of the wild type tomato (*Solanum lycopersicum* (Mill. cv. Ailsa Craig AC^++^)) and ripening mutants (*Nr* and *rin*). Low expression levels of *SlCMB1* were observed in roots, leaves and B (Breaker) to B + 7 (Breaker + 7d) fruits (Fig. 2A). Whereas, predominant expression was observed in flowers, stems, IMG and MG fruits (Fig. 2A,B), suggesting that *SlCMB1* might play roles in the development of these tissues. Furthermore, the transcripts of *SlCMB1* rapidly declined with fruit ripening in AC^++^ fruits, and a simlar expression pattern of *SlCMB1* was observed in the mutant *Nr* and *rin* fruits (Fig. 2A,B). But the expression levels of *SlCMB1* in *Nr* and *rin* fruits at IMG and MG stages were significantly less than in AC^++^ (Fig. 2B), suggesting that *SlCMB1* expression may be impacted by *SlMADS-RIN* and/or ethylene(Fig. 2B).

### Generation of *SlCMB1*-RNAi Lines

**Figure 3:**
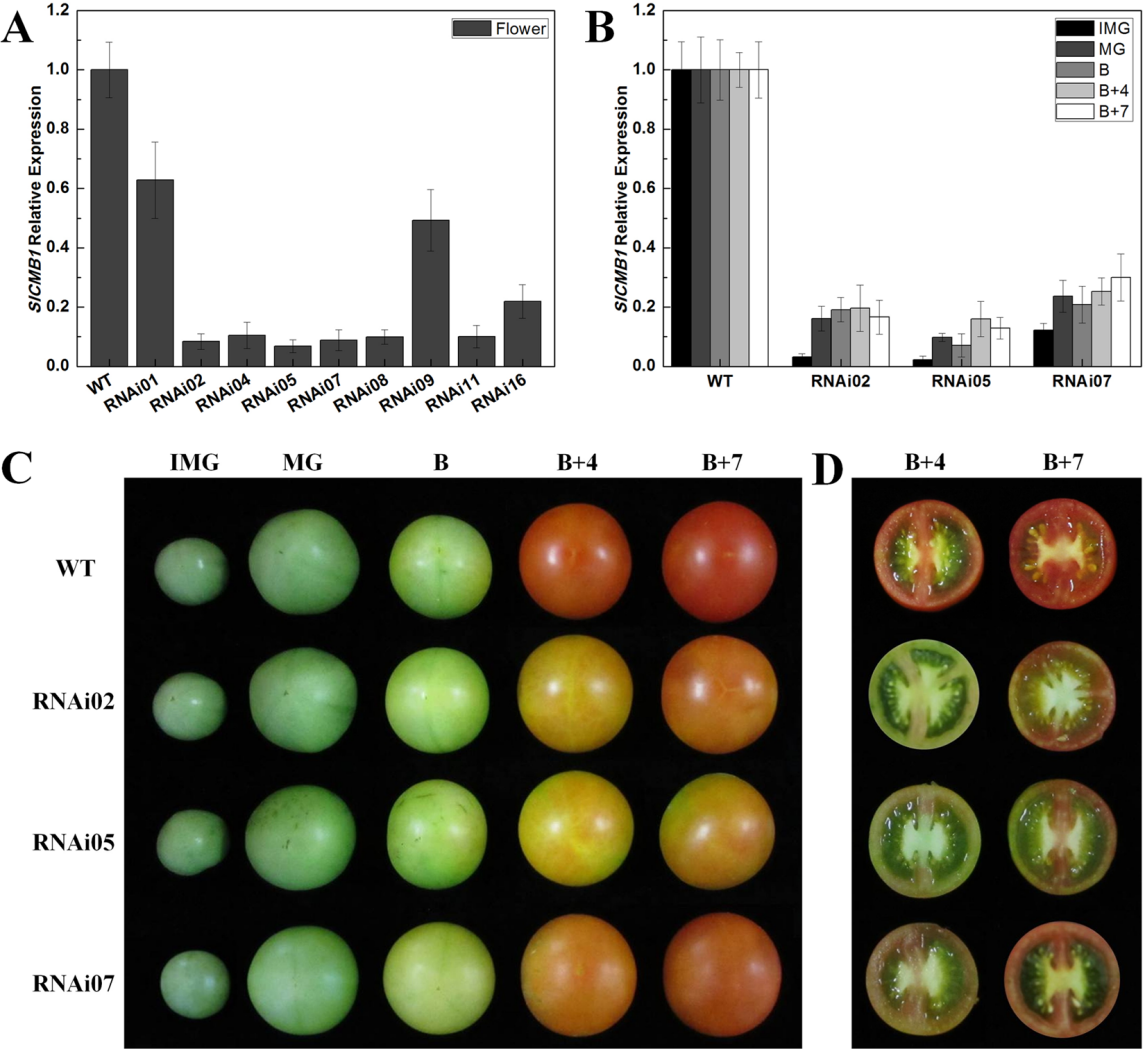
Phenotypes and expression analyses of *SlCMB1* in RNAi lines. (**A**) The relative expression of *SlCMB1* between the WT and all nine *SlCMB1* RNAi lines. The tissue is flower at anthesis. (**B**) Relative expression of *SlCMB1* in the WT and the three selected silencing lines from IMG to B + 7 fruits. The tissues are the fruits at different stages (**C**). Phenotypes of fruits at different stages from IMG to B + 7 stage in WT and RNAi lines. (**D**) Transverse sections of WT and *SlCMB1* RNAi fruits at the B + 4 and B + 7 stage. WT, wild type. Data are the means ± SD of three independent biological replicates. The transcripts of *SlCMB1* in the wild type are normalized to 1. Error bars indicate SE.

To further study the roles of *SlCMB1* in tomato growth and development, an RNAi expression vector targeting the C-terminal specific fragment of *SlCMB1* was generated (Supplementary Fig. S2) and transferred into the wild type tomato (AC^++^). Nine independent transgenic lines were confirmed by PCR with primers of *NPT II* (Supplementary Table S1), then total RNAs of these independent transgenic lines were extracted from flowers to investigate the relative expression of *SlCMB1*, respectively. The qRT-PCR results displayed that the transcript levels of *SlCMB1* in six transgenic lines were significantly decreased by 90–94% compared with the wild-type (Fig. 3A). Later, three independent transgenic lines (RNAi02, RNAi05, RNAi07) which had the lowest accumulation of *SlCMB1* transcripts were selected for further characterization.

### Expression profile analysis of ripening- and carotenoid-related genes in *SlCMB1*-RNAi fruits

**Figure 4:**
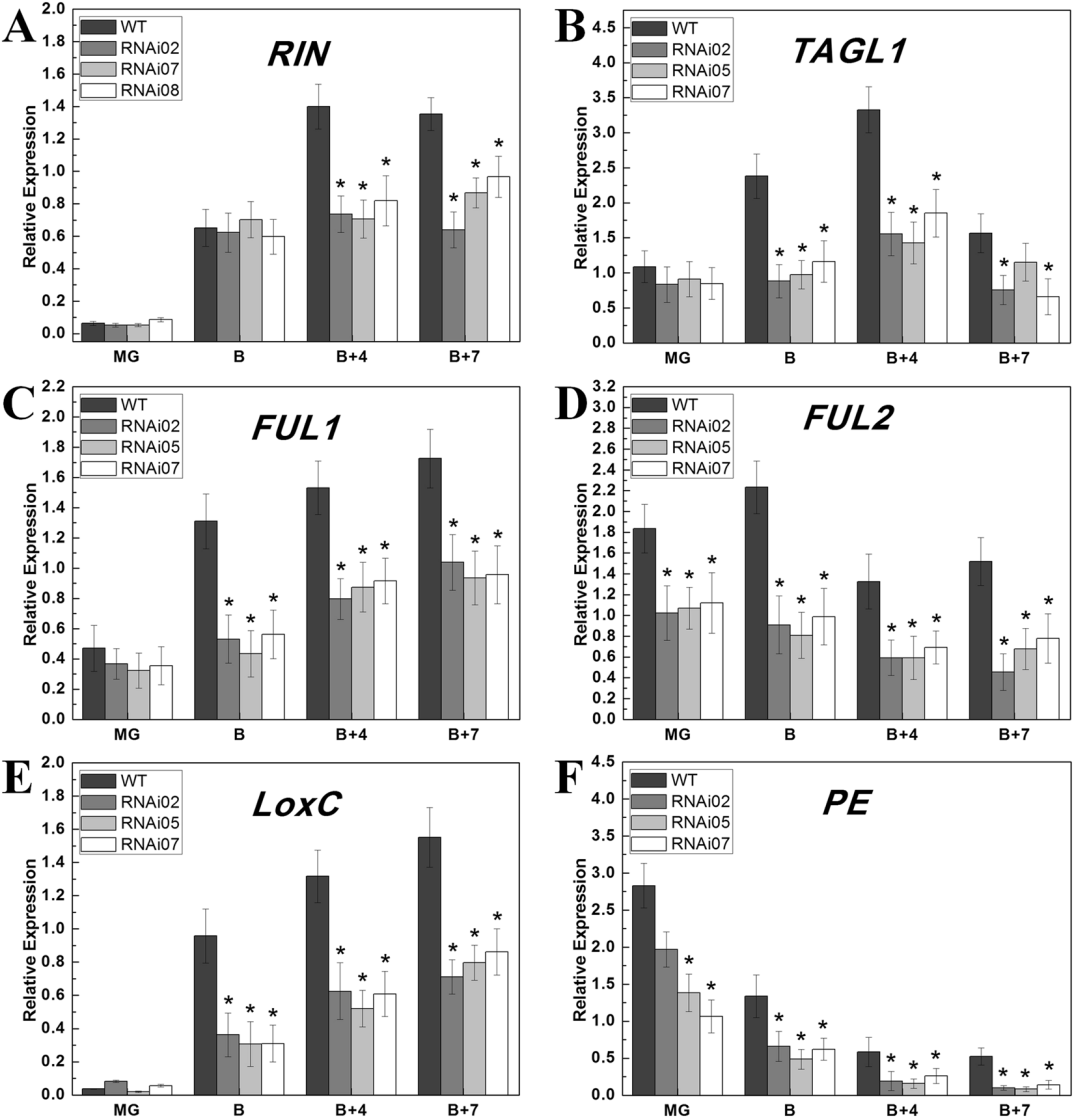
Relative expression of ripening-related genes in fruits of the wild type (WT) and *SlCMB1*-RNAi lines. The total RNA were extracted for the qRT-PCR assay from MG, B, B + 4 and B + 7 fruits of WT and RNAi lines. Three independent biological replications of each sample were used. (**A**) Expression of *RIN* in RNAi lines and the wild type. (**B**) Expression of *TAGL1* in RNAi lines and the wild type. (**C**) Expression of *FUL1* in RNAi lines and the wild type. (**D**) Expression of *FUL2* in RNAi lines and the wild type. (**E**) Expression of *LoxC* in RNAi lines and the wild type. (**F**) Expression of *PE* in RNAi lines and the wild type. MG, mature green; B, breaker; B + 4, 4 days after B; and B + 7, 7 days after B. The significant differences were marked with the asterisks between the RNAi and WT fruits (P < 0.05). Error bars indicate SE.

To verify the suppression of *SlCMB1* in the selected transgenic lines, the total RNA was extracted from IMG, MG, B, B + 4 and B + 7 fruits of WT and the transgenic lines, respectively. The qRT-PCR result displayed that the transcripts of *SlCMB1* in fruits at different stages of selected transgenic lines (RNAi02, RNAi05, RNAi07) were markedly reduced to roughly 5–30% of control levels (Fig. 3B). To confirm the specific suppression of *SlCMB1*, the expression of *RIN* was tested, because it is the most closely related (67.3% identity at nucleotide level) gene to *SlCMB1* in tomato. Meanwhile, the result of multiple sequence alignment between *RIN* and *SlCMB1* fragment showed that the 426 bp fragment of *SlCMB1* using in our study was specific (Supplementary Fig. S3). Figure 4A showed that there was no significant difference of *RIN* expression in *SlCMB1*-RNAi fruits and the wild type at MG and B stage but its transcripts were reduced significantly at B + 4 and B + 7 stage in *SlCMB1*-RNAi fruits. These results indicated that the reduced expression of *RIN* at B + 4 and B + 7 stage of transgenic fruits was not caused by the construct of *SlCMB1*-RNAi vector but the suppression of *SlCMB1* in tomato fruits.

Because the ripening time of *SlCMB1*-RNAi fruits was delayed 3 to 5 days (Table 1), so the expression of a number of known ripening-related genes were analyzed in *SlCMB1*-RNAi and the wild-type tomato fruits. The results showed that the transcripts of five ripening-related genes, *RIN*, *TAGL1*, *FUL1*, *FUL2*, *LoxC* (*Lipoxygenase C*) and *PE* (*PECTINESTERASE*) were significantly down-regulated in *SlCMB1*-RNAi fruits (Fig. 4A–F). These results suggested that *SlCMB1* might regulate the fruit ripening of tomato through influencing the expression of ripening-related genes.

**Table 1:** Days from anthesis to B stage for the wild type and *SlCMB1*-RNAi lines.

Tomato Lines	Days
Wild type	37.9 ± 0.66
RNAi02	42.1 ± 0.68
RNAi04	41.1 ± 0.82
RNAi05	43.3 ± 0.51
RNAi07	41.8 ± 0.73
RNAi08	41.4 ± 0.68

**Figure 5:**
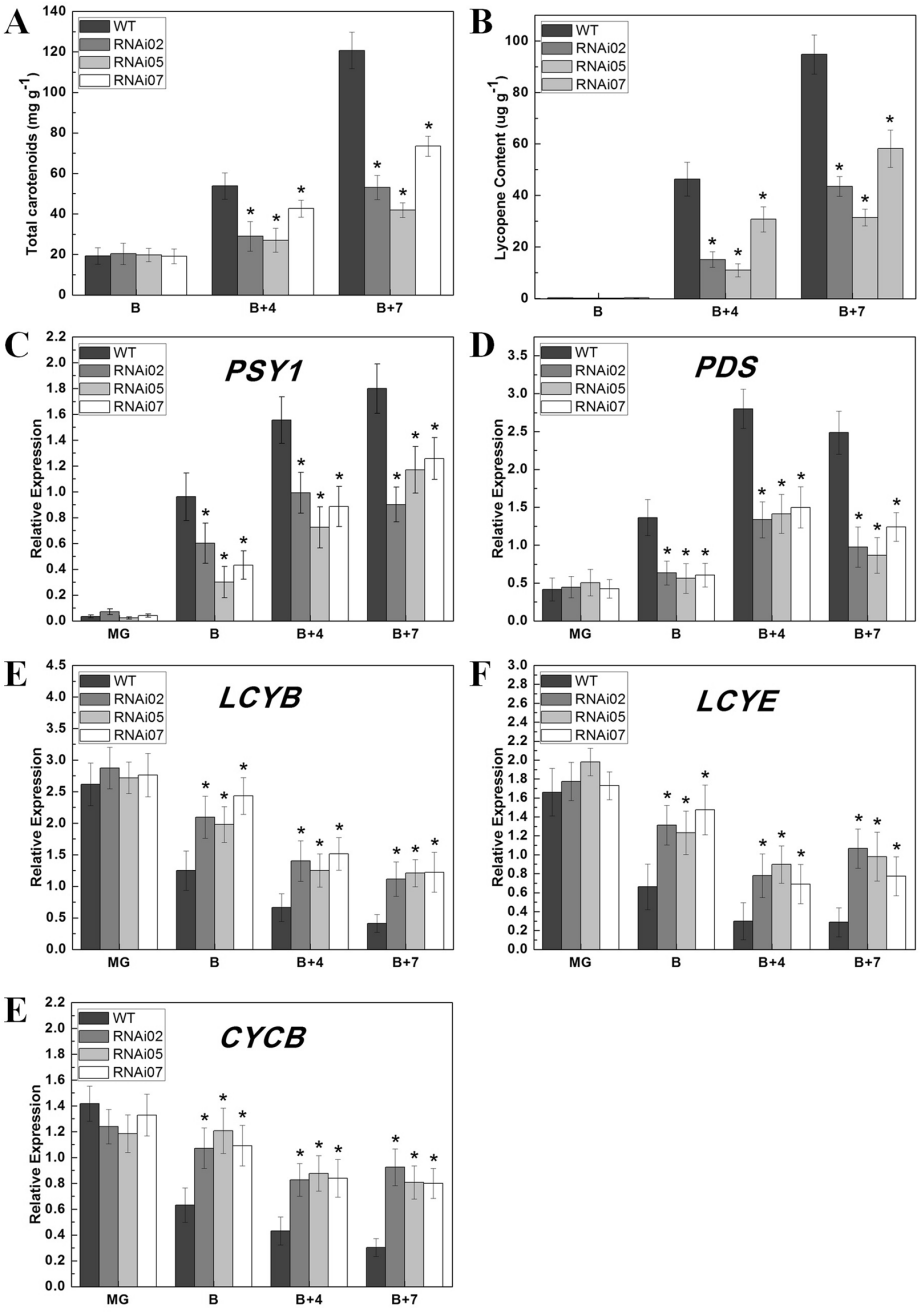
Pigments accumulation and relative expression levels of the carotenoid synthesis related genes in the *SlCMB1* RNAi and the wild-type (WT) fruits. (**A**) Analysis of carotenoid accumulation in B, B + 4 and B + 7 fruits of the *SlCMB1*-RNAi lines and the wild type. (**B**) Analysis of lycopene content in B, B + 4 and B + 7 fruits of the *SlCMB1*-RNAi lines and the wild type. (**C**) Expression of *PSY1* in fruits of the wild type and transgenic lines. (**D**) Expression of *PDS* in fruits of the wild type and transgenic lines. (**E**) Expression of *LCYB* in fruits of the wild type and transgenic lines. (**F**) Expression of *LCYE* in fruits of the wild type and transgenic lines. (**G**) Expression of *CYCB* in fruits of the wild type and transgenic lines. MG, maturate green; B, breaker; B + 4, 4 days after B; B + 7, 7 days after B. Three independent biological repeats of each sample were used. Data are presented as means ± SD of at least three individual fruits. The significant differences were marked with the asterisks between the RNAi and WT fruits (P < 0.05). Error bars indicate SE.

For the reduced carotenoid accumulation was observed in transgenic fruits, several known genes which have been reported to be involved in the carotenoid biosynthesis pathway were examined. It has been reported that *PSY1* is the rate-limiting enzyme in the synthesis process of lycopene and is induced by ethylene^50^. In this study, the qRT-PCR results showed that its expression was dramatically down-regulated in B, B + 4 and B + 7 fruits of the *SlCMB1*-RNAi lines (Fig. 5B). Another enzyme, phytoene desaturase (PDS), which involved in the lycopene synthesis was also notablely down-regulated in *SlCMB1*-RNAi lines (Fig. 5C). Furthermore, other three lycopene cyclase genes, *CYCB* (chromoplast-specific lycopene cyclase), *LCYB* (lycopene β-cyclase) and *LCYE* (lycopene ε-cyclase), which participate in the cyclization of lycopene were significantly up-regulated in the *SlCMB1*-RNAi tomato fruits at different stages (Fig. 5D–F). These results indicate that suppression of *SlCMB1* reduced the carotenoid accumulation and influenced the transcripts of related genes involving in the carotenoid biosynthesis pathway.

### Silencing of *SlCMB1* inhibited fruit ripening and carotenoid accumulation

In the process of tomato fruit development and ripening, the time from pollination to fruit ripening was recorded. We found that the red color of wild type fruit was deeper than the *SlCMB1*-silenced fruit (Fig. 3C,D) and the ripening time of *SlCMB1*-silenced fruits was delayed 3 to 5 days as compared to the wild type (Table 1). Previous studies have reported that the accumulation of carotenoids is the main reason for the pigmentation change in tomato ripening fruits^51^. In our study, we extracted and examined the total carotenoids and lycopene in B, B + 4 and B + 7 fruits of the RNAi lines and the wild-type. As shown in Fig. 5A,B, the carotenoid and lycopene contents in B + 4 and B + 7 fruits of *SlCMB1*-RNAi lines were significantly lower than in the wild type tomato plants, indicatng that reduction of *SlCMB1* transcripts inhibited fruit ripening and altered the carotenoid content of tomato fruits.

### Repression of *SlCMB1* reduced the production of ethylene and the transcripts of ethylene-related genes

**Figure 6:**
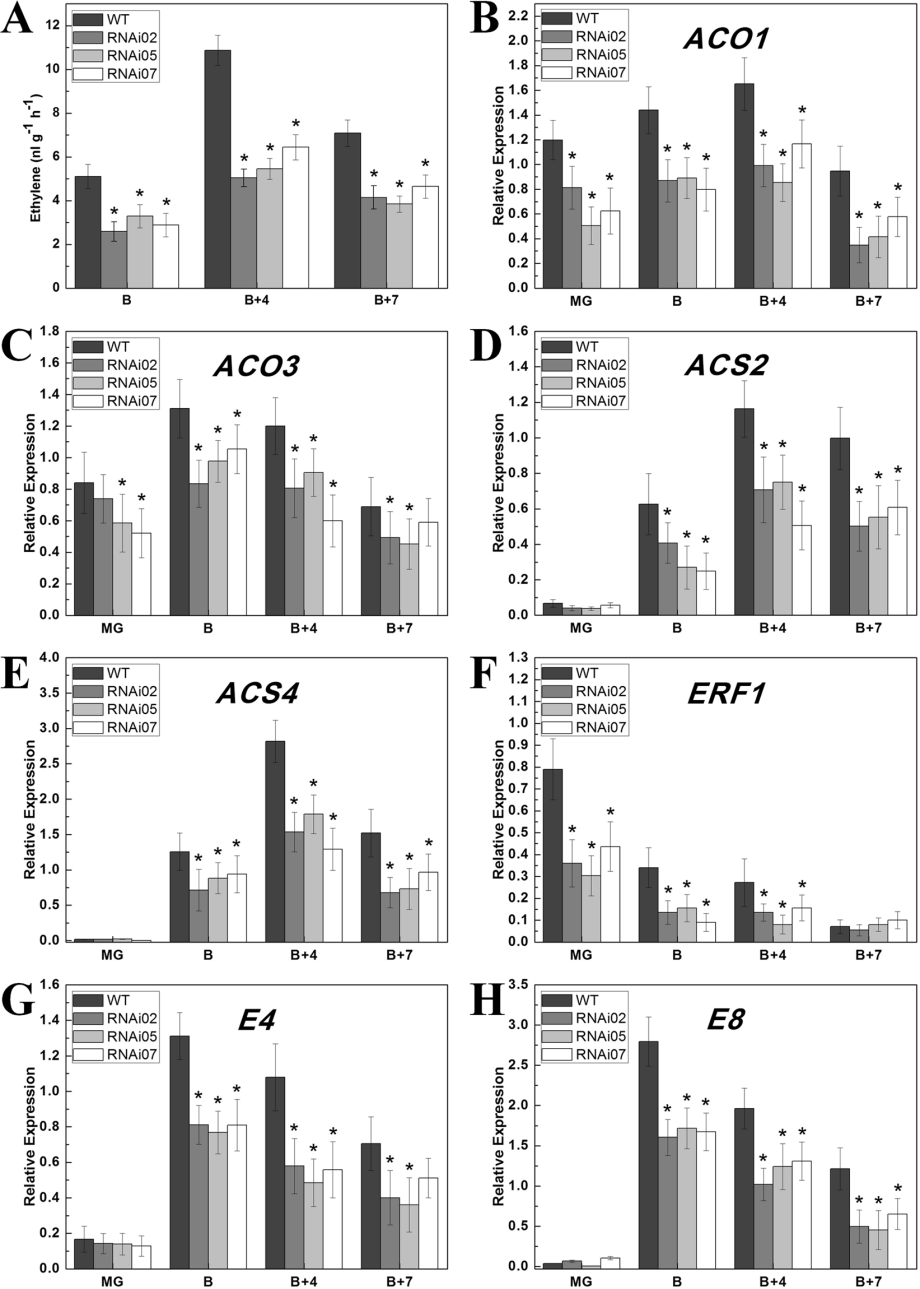
Determination of ethylene production and the expression of ethylene-related genes in the wild type (WT) and the *SlCMB1*-RNAi fruits. (**A**) Production of ethylene in WT and *SlCMB1*-RNAi fruits. B, B + 4 and B + 7 fresh fruits were sealed in air-tight jars and then the 1 mL headspace gas was sampled 24 h later. The date represent means from at least three biological repeats. (**B**–**E**) Expression of four ethylene biosynthesis-related genes (*ACO1*, *ACO3*, *ACS2* and *ACS4*) in fruits of *SlCMB1*-RNAi and the wild type. (**F**–**H**) Expression of three ethylene responsive genes (*ERF1*, *E4* and *E8*) in WT and *SlCMB1* RNAi fruits. The total RNA were extracted for qPCR assay from MG (mature green), B (breaker), B + 4 (4 days after B) and B + 7 (7 days after B) fruits of WT and RNAi lines. Three replications for each sample were used. The significant differences were marked with the asterisks between the RNAi and WT fruits (P < 0.05). Date are the means ± SD of three independent biological replicates. Error bars indicate SE.

Fruit ripening and carotenoid accumulation of tomato were affected by the gas hormones ethylene^52^. To further explore the impacts of reduced *SlCMB1* mRNA on ethylene biosynthesis, ethylene production was measured during the process of fruit ripening. The result showed that the ethylene production of wild-type fruits displayed a massive and rapid accumulation at B + 4 stage, and declined at B + 7 stage. *SlCMB1*-RNAi fruits exhibited a similar trend in production of ethylene similar to the wild type with the highest level of ethylene in the B + 4 stage. However, overall ethylene production in *SlCMB1*-RNAi fruits were approximately 50% lower when compared to the wild type in all stages (Fig. 6A).

The ethylene production was affected by the transcripts of the ethylene synthesis and response genes. Given that the reduction of ethylene production in the *SlCMB1*-RNAi lines, multiple crucial genes involved in the ethylene biosynthesis and response were detected in fruits of the wild-type and RNAi lines at different stages. The results showed that four crucial ethylene biosynthesis genes (*ACO1*, *ACO3*, *ACS2* and *ACS4*) and three ethylene response genes (*E4*, *E8* and *ERF1*) were notably down-regulated in *SlCMB1*-RNAi fruits, especially in B and B + 4 fruits (Fig. 6B–H). These results suggested that down-regulation of *SlCMB1* reduced the ethylene production and the transcripts of ethylene biosynthesis and response genes.

### SlCMB1 could interact with SlMADS-RIN, SlMADS1, SlAP2a and TAGL1

**Figure 7:**
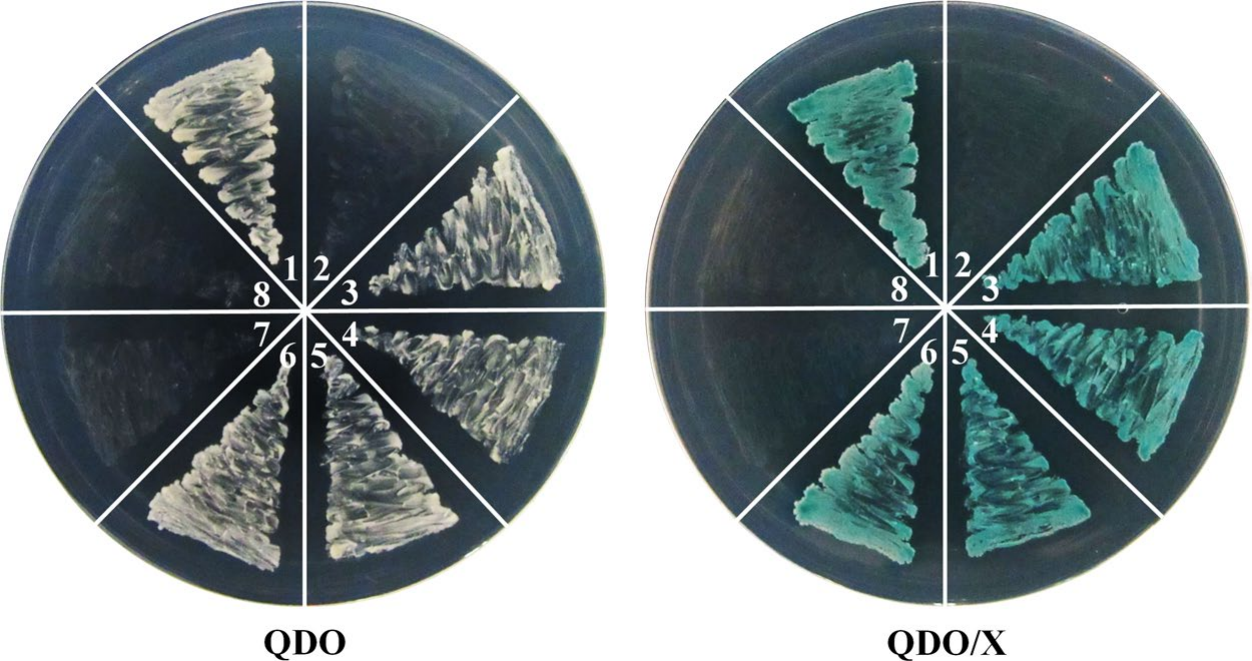
Yeast two-hybrid assay of SlCMB1 with SlMADS-RIN, SlMADS1, SlAP2a and TAGL1. QDO, SD medium lacking Trp, Leu, His, and adenine. QDO/X, SD medium lacking Trp, Leu, His, and adenine with X-a-Gal. 1. pGBKT7-53 & pGADT7-T (positive control); 2. pGBKT7-Lam & pGADT7-T (negative control); 3. pGADT7-SlCMB1 & pGBKT7-RIN; 4. pGADT7-SlCMB1 & pGBKT7-SlMADS1; 5. pGADT7-SlCMB1 & pGBKT7-SlAP2a; 6. pGADT7-SlCMB1 & pGBKT7-TAGL1; 7. pGADT7 (empty bait vector control); 8. pGBKT7 (empty prey vector control).

To confirm the existence of interaction between SlCMB1 and other ripening-related proteins, SlMADS-RIN, SlMADS1, SlAP2a and TAGL1 were preferentially selected for the yeast two hybrid assays. The ORF (open reading frames) of these four genes were amplified, respectively. The ORF of *SlCMB1* was cloned into the pGADT7 vector to be as the prey and the ORF of *SlMADS-RIN*, *SlMADS1*, *SlAP2a* and *TAGL1* was cloned into the pGBKT7 vector to be as the bait, respectively. Self activation of pGBKT7-RIN, pGBKT7-SlMADS1, pGBKT7-SlAP2a and pGBKT7-TAGL1 were tested and the results were negative (Supplementary Fig. S5). Furthermore, the empty prey and bait vector containing the construct of each prey and bait were used as the negative controls, respectively. Figure 7 displayed that the yeast could grow on the selective medium (QDO) and turn blue on the plate containing the X-α-gal indicator (QDO/X), indicating that SlCMB1 can interact with SlMADS-RIN, SlMADS1, SlAP2a and TAGL1, respectively.

## Discussion

In this study, we characterized a SEP MADS-box gene, *SlCMB1*, which is recently reported to be involved in the regulation of tomato (*S*. *pimpinellifolium*) inflorescence architecture^48^ and named it following the existing name on the NCBI web site. It is known that MADS-box genes always play diverse roles in different developmental processes, such as *MC* (*MACROCALYX*), *J* (*JOINTLESS*) and *SlMBP21* and so on^48,53–57^. In order to further investigate other potential roles of *SlCMB1* in the development of tomato, we isolated this gene from *Solanum lycopersicon* (Mill. cv. Ailsa Craig) and investigated this gene. SlCMB1 had a higher homology with RIN, the key regulator of fruit ripening, at the amino acid level. The promoter analysis result displayed that the ethylene-responsive element, ERE-motif (ATTTCAAA) (Supplementary Fig. S1)^58,59^, was found in the promoter region of *SlCMB1* gene. Expression profile of *SlCMB1* showed that its transcripts rapidly declined with fruit ripening in the wild type fruits, and its expression pattern in *Nr* and *rin* mutant fruits was similar with that in AC^++^ fruits (Fig. 2A,B). These results indicated that *SlCMB1* may involve in fruit ripening and/or ethylene biosynthesis.

In plants, ethylene plays a very important role in fruit ripening, and the biosynthesis pathway of ethylene has been well studied^60–62^. Two patterns of ethylene production systems, the autoinhibitory (system 1) and the autocatalytic (system 2), have been identified. In the system 1, basal ethylene is produced in the immature fruits and vegetative organs, while in the system 2 ethylene production is greatly increased at the beginning of flower senescence and climacteric fruit ripening^17,63^. ACS (ACC synthase) and ACO (ACC oxidase) are the critical rate-limiting enzymes in the process of ethylene biosynthesis. In tomato, co-suppression of *SlACS2* and *SlACS4* using antisense approach could reduce the ethylene biosynthesis of system 2 and the fruit ripening is inhibited in the transgenic lines^64^. In addition, *SlACS2* plays a significant role in the transition of ethylene biosynthesis system (from system 1 to system 2)^17^. It has been reported that *SlACO1* and *SlACO3* express at the beginning of fruit ripening, the transcripts peak of *SlACO1* at B + 3 stage and then falls back to B stage levels, whereas *SlACO3* expresses transiently during ripening, the synthesis of ACO1 may be the first step of ethylene synthesis, after that the transcripts of ACS genes are induced by produced ethylene and then more ACC are produced^18^. In our study, the transcripts of *SlACS2*, *SlACS4*, *SlACO1* and *SlACO3* were significantly decreased in *SlCMB1*-silenced fruits (Fig. 6B–E). Moreover, the production of ethylene in *SlCMB1*-silenced fruits is significantly lower than the wild type (Fig. 6A). These results suggested that *SlCMB1* may contribute to ethylene biosynthesis by promoting the expression of ethylene synthesis genes during tomato fruit ripening.

It has been known that *E4*, *E8* and *ERF1* are important ethylene-responsive genes in the process of fruit ripening^23,25^. In the mutations which block fruit ripening, the transcript of *E4* is suppressed with the inhibited high-level ethylene biosynthesis^23^. *E8* is reported to involve in ethylene biosynthesis in the process of fruit ripening^25^. The ethylene-responsive gene *ERF1* which is initiated by ethylene is reported as an immediate target for *EIN3*^65^. In this study, the transcripts of these three genes (*E4*, *E8* and *ERF1*) were dramatically reduced in the *SlCMB1*-RNAi fruits compared with the wild type (Fig. 6F–H). Down-regulation of these ethylene response genes indicated that suppression of *SlCMB1* impacts ethylene synthesis and fruit ripening.

Previous studies showed that four MADS-box proteins, RIN, FUL1, FUL2, and TAGL1, are essential to fruit ripening^40,43,66^. Recently, the molecular and biochemical studies indicate that *RIN* involves in ethylene biosynthesis by increasing the transcripts of ethylene biosynthesis genes and ethylene signaling genes such as *ACS2* and *ACS4*^36,67,68^. Further more, *RIN* could directly regulate the expression of *FUL1* by binding to its promoter^69^ and another MADS-box gene, *TAGL1*, which activity in ripening is executed through direct activation of *ACS2* that is reported to be a target of MADS-RIN^36,70^. In this study, the expression levels of these four MADS-box genes, *RIN*, *TAGL1*, *FUL1* and *FUL2*, were down-regulated in the *SlCMB1*-RNAi fruits (Fig. 4A–D). *RIN* is the key regulator in tomato fruit ripening and acts upstream of ethylene biosynthesis, when it is repressed, fruit ripening and ethylene biosynthesis will be delayed^32^. In this study, although there was no significant difference of *RIN* expression in *SlCMB1*-RNAi fruits at MG and B stage, the remarkable reduced *RIN* mRNA in tomato plants were observed in transgenic fruits at B + 4 and B + 7 stage (Fig. 4A) suggesting that there might be direct or indirect regulatory relationship between *SlCMB1* and *RIN*. In addition to these genes described above, the transcripts of other one ripening-related gene, *LoxC*, and a cell wall metabolism gene, *PE*, were also inhibited in *SlCMB1*-RNAi fruits (Fig. 4E,F). Moreover, phenotype analysis showed that fruit ripening in *SlCMB1*-silenced lines was delayed (Fig. 3C,D; Table 1). These results indicated that repression of *SlCMB1* impacts the transcripts of ripening-related genes and delays the fruit ripening.

In this study, suppression of *SlCMB1* resulted in delayed fruit ripening (Fig. 3C,D, Table 1) and reduced ethylene production (Fig. 6A). This is an interesting and new finding about this gene after it is reported to be involved in the regulation of inflorescence development^48^. This new finding will be contribute to helping us to further study the roles of *SlCMB1* in the process of tomato growth and development. Up to now, a number of MADS-box genes have been reported to play multiple roles in the growth and development of plants, such as *SlMADS1*, *SlMBP21* and *TAGL1*^41,48,54,55,70–74^. For example, *SlMADS1* (*Solyc03g114840*) was recently reported to play essential roles in the inflorescence development of tomato after it was found to be as a negative regulatory factor in tomato fruit ripening^46,48^. Further more, co-repression of the tomato FRUITFULL homologues (*FUL1* and *FUL2*) results in the delayed fruit ripening and the decreased transcripts of ripening- and ethylene-related genes^43^. Similar alteration of fruit ripening is also observed in *rin*, *Nr*, *nor*, and *Cnr* mutant: delayed fruit ripening and reduced ethylene production^29,32,33,35^. According to our results and previous studies, we can speculate that *SlCMB1* may be involved in the regulation of ethylene synthesis and fruit ripening, possibly through affecting the expression of ethylene synthesis, ethylene response and ripening-related genes.

The red pigmentation in tomato ripening fruits is mainly made up of β-carotene (5–40%) and lycopene (70–90%) which represent most of total carotenoids conferring the orange color and the red color, respectively^75,76^. Up to now, it is well known that the defects of carotenoid biosynthesis results in the reduction of carotenoid accumulation in the ripening-deficient mutant fruits^77^. The decreased expression of *SlCMB1* leads to dramatically reduced carotenoid and lycopene content (Fig. 5A,B), explaining partly why *SlCMB1*-silenced fruits do not completely turn red at the same stage compared with the wild type (Fig. 3C,D). Moreover, the displayed orange-yellow or orange *SlCMB1*-silenced fruits (B + 4 and B + 7) and decreased carotenoid and lycopene content also implies increased β-carotene accumulation.

It is reported that PSY1, PDS, LCYE, LCYB and CYCB are the major enzymes and PSY1 is the rate-limiting enzyme in the carotenoid biosynthesis pathway. PSY1 catalyzes the conversion of geranylgeranyl diphosphate (GGPP) to phytoenethe, and PDS catalyzes the conversion of phytoene to ζ-carotene. In the carotenoid biosynthetic pathway, cyclization of lycopene forms two branches: one branch results in β-carotene and xanthophylls which catalyzed by two chloroplast and chromoplast lycopene β-cyclases, LCYB and CYCB, and the other results in α-carotene and xanthophyll catalyzed by LCYE and LCYB^78^. Furthermore, CYCB, a major enzyme in the cyclization of lycopene, is reported to be responsible for the transition from lycopene to β-carotene^79^. It is reported that the relative content of β-carotene and lycopene in tomato fruits during normal ripening is mediated by increased *PSY1* transcripts and reduced expression of *CYCB*, in which these two effects are regulated partly by ethylene due to the induction of ethylene to *PSY1*^51,79,80^. In this study, the expression of *PSY1*, which is induced by ethylene and is a crucial regulator of carotenoids biosynthesis during fruit ripening, was significantly down-regulated in response to reduced *SlCMB1* (Fig. 5B). *PDS*, another regulator of carotenoids biosynthesis, was also notably inhibited in transgenic fruits (Fig. 5C), whereas the transcripts of *CYCB*, *LCYB* and *LYCE* were significantly increased in *SlCMB1*-RNAi fruits compared to the wild type (Fig. 5D–F). The change in expression of these genes is consistent with the decreased carotenoid and lycopene content. Previous studies have shown that RIN could interact with the major limiting enzyme PSY1 which is a direct target of RIN to control the pigment accumulation in carotenoid pathways during fruit ripening^36,69^. In this study, the expression of *RIN* was suppressed in the fruits of *SlCMB1*-RNAi lines at B + 4 and B + 7 stage (Fig. 4A) and the yeast two-hybrid assay showed that SlCMB1 can interact with SlMADS-RIN, SlMADS1, SlAP2a and TAGL1, respectively (Figs 7; S5). These results can explain, on molecular and protein level, why the *SlCMB1*-silenced fruits exhibited a kind of light orange or orange phenotype at B + 4 and B + 7 stage (Fig. 3C,D). Analogously, previous studies showed that repression of the MADS-box gene *TAGL1* leads to reduced ethylene production and increased β-carotene accumulation during fruit ripening^40^. Moreover, suppression of a tomato *AP2*/*ERF* gene, *SlAP2a*, results in reduced carotenoid accumulation through altering carotenoid pathway flux^81,82^. Based on previous investigations, we could speculate that *SlCMB1* may play a significant role in regulation of carotenoid synthesis away from the lycopene and flux toward the β-carotene in *SlCMB1*-RNAi fruits, possibly through impacting ethylene biosynthesis or signal transduction or through regulating the expression of *PSY1* by interacting with SlMADS-RIN.

Recent years, a growing number of transcription factor family, especially the MADS-box transcription factors, have been characterized and identified to play important regulatory roles in fruit ripening. It has been reported that MADS-box proteins can form homodimers, heterodimers, or higher-order protein complexes with other proteins to regulate plant growth and development^83–85^. Among the MADS-box proteins, SlMADS-RIN, a classical regulatory factor of fruit ripening, involves in the ethylene synthesis, ethylene response and ethylene perception in tomato^66^. Previous reports have shown that SlMADS-RIN can bind to SlACS2 and SlACS4 and associates with their promoters^36,67,69^. What is more, SlMADS-RIN also indirectly influences *SlACO1* expression through binding to the promoter of a homeobox gene, *HB-1*, which generates an interaction with the promoter of *SlACO1*^36,86^. Recent studies have shown that the ethylene-responsive genes *E8* which could be induced by ethylene in fruit ripening is reported to be the direct target of SlMADS-RIN^36,87^. In addition, *SlMADS1*, *SlAP2a* and *TAGL1* are the significant regulators in the process of fruit ripening. The MADS-box protein, SlMADS1, is reported to inhibit ethylene biosynthesis and influences fruit ripening as a negative regulator by interacting with SlMADS-RIN^46^. When *SlAP2a*, a member of the AP2/ERF superfamily, was repressed by RNAi approach in tomato, the transgenic lines displayed shorter ripening time and altered carotenoid accumulation^81,82^. *TAGL1*, another MADS-box gene, is reported to be as a positive regulator to involve in the regulation of fruit ripening and fleshy fruit expansion and suppression of *TAGL1* in tomato results in yellow-orange fruits with thiner pericarps, decreased carotenoids and delayed ripening^40,70^. In our study, the yeast two-hybrid assays showed that SlCMB1 could interact with SlMADS-RIN, SlMADS1, SlAP2a and TAGL1, respectively (Figs 7; S5). Previous studies have reported that the tomato FRUITFULL homologues (FUL1 and FUL2) act in fruit ripening via forming heterodimers with MADS-RIN^43^. The yeast three-hybrid assays have displayed that FUL1, TAGL1 and RIN could form higher order complexs^88^. Wang, S. *et al*. thought that there might be higher order complexes between FUL1, FUL2, MADS-RIN and TAGL1 in tomato fruit ripening^89^. Similarly, higer order complexs may also exists among SlCMB1, SlMADS-RIN, SlMADS1, SlAP2a and TAGL1 in the process of tomato fruit ripening. So we can speculate that SlCMB1 may increase the activity of SlMADS-RIN and TAGL1 and/or reduce the activity of SlMADS1 and SlAP2a through forming dimers or higher-order protein complexes to directly or indirectly regulate the expression of related genes such as *ACO1*, *ACS2*, *ACS4* and *E8*, and finally the ethylene biosynthesis is increased and the fruit ripening is promoted.

In summary, the MADS-box transcription factor *SlCMB1* plays an essential role in the process of fruit ripening acting as a positive regulator by modulating the ethylene biosynthesis and response and carotenoid accumulation through interacting with SlMADS-RIN, SlMADS1, SlAP2a and TAGL1. *SlCMB1* is a new member of the regulatory network of fruit ripening. Additionally, our results manifest that the higher levels of the *SlCMB1* regulatory cascades in tomato fruit ripening await being discovered, such as identification of upstream regulatory factors, direct or indirect downstream targets and the interaction between these new regulatory components. We can believe that these follow-up works will contribute to adding more new components to enrich the ripening regulatory network and will bring a deeper understanding to the fruit ripening regulatory mechanism.

## Materials and Methods

### Plant materials and growth conditions

In this study, the near-isogenic tomato line, *Solanum lycopersicum* (Mill. cv. Ailsa Craig AC^++^), was used as the wild type. The wide type and transgenic tomato plants were planted in the greenhouse under the standard conditions as follows: 25 °C for 16 h (day) and 18 °C for 8 h (night). The tomato flowers were tagged at anthesis. The days and fruit color post-anthesis (DPA) was used to differentiate the ripening days of tomato fruits. In the wild type, we defined 20 DPA as the immature green, 35 DPA as the mature green that the fruit is green and shiny and no obvious color change is observed. The 38 DPA tomato fruits which color of fruits change from green to yellow was characterized as breaker (B) fruits. Besides, the material of B + 4 (4 days after breaker) fruits and B + 7 (7 days after breaker) fruits were also used in our study. All the needed samples were collected and immediately frozen in liquid nitrogen and then stored at −80 °C until being used.

### Total RNA extraction, isolation and sequence analysis of *SlCMB1*

The total RNA from all WT and transgenic tomato plants tissues was extracted using the RNAiso Plus reagent (Takara, China) following the instructions of manufacturer. In order to synthesize the first strand cDNA, 1 μg total RNA samples which were digested with the Dnase I (Promega, USA) was used to perform the reverse transcription using the M-MLV reverse transcriptase (Promega) with the oligo(dT)_20_ primer according to the manufacturer’s protocol.

The full length of *SlCMB1* gene was cloned using 1–2 μL cDNA with primers *SlCMB1-Full-F* and *SlCMB1-Full-R* (Supplementary Table S1). The DNA A-Tailing kit (Takara) was used to tail the amplified products. After that, the tailed products were cloned into the pMD18-T vector (Takara). The *Escherichia coli* JM109 transformation was performed to pick out the positive clones and confirmed by sequencing. Multiple sequence alignment was performed for comparison with other MADS-box proteins by DNAMAN (Version 6.0). The phylogenetic tree was constructed by MEGA(Version 5.2) according to the neighbor-joining bootstrap method as follows: bootstrap analysis of 1,000 replicates, pairwise deletion and poisson model. Moreover, 3500 bp nucleotide sequence upstream of the initiation codon ATG of predicted ORF of *SlCMB1* gene was used to perform the promoter analysis on the Plant CARE website (http://bioinformatics.psb.ugent.be/webtools/plantcare/html/).

### RNAi vector construction of *SlCMB1* and plant transformation

In order to study the function of *SlCMB1* in the process of tomato fruits development, a RNAi vector of *SlCMB1* was constructed to down-regulate its expression. A 426-bp 3′ special fragment of *SlCMB1* was amplified using primers *SlCMB1-i-F* and *SlCMB1-i-R* (Supplementary Table S1) which was tailed with the *Kpn*I/*Hin*dIII and the *Xho*I/*Xba*I restriction enzyme sites at the 5′ end of primers, respectively. The enzyme digestion was performed using the *Hin*dIII/*Xba*I and *Kpn*I/*Xho*I enzyme to digest the amplified *SlCMB1* fragments, respectively. Then the digested products were ligated into the sense orientation at the *Hin*dIII/*Xba*I restriction enzyme site and into the antisense orientation at the *Kpn*I/*Xho*I restriction enzyme site of pHANNIBAL plasmid, respectively. After being digested with *Spe*I/*Sac*I, the expression unit what we needed, including the 35 S promoter of the cauliflower mosaic virus, the specific fragment of *SlCMB1* in the antisense orientation, the PDK intron, specific fragment of *SlCMB1* in the sense orientation, and the OCS terminator, was subcloned into the pBIN19 vector at the *Sac*I and *Xba*I restriction site to obtain the RNAi vector for *SlCMB1* gene silencing (Supplementary Fig. S2).

The generated binary plasmids which were verified by restriction digest analysis and by sequencing were transferred into the *Agrobacterium tumefaciens* LBA4404 strain, and *Agrobacterium tumefaciens*-mediated transformation was carried out according to the approach described by Chen *et al*.^90^. The primers *NPTII*-F and *NPTII*-R (Supplementary Table S1) were used to detect the transgenic plants. The selected positive transgenic lines were used for the subsequent investigation.

### Quantitative real-time PCR (qRT-PCR) analysis

Total RNA extraction and reverse transcription were performed as described above. Then 5 times RNase/DNase-free water was used to dilute the synthesized cDNAs for the qRT-PCR analysis. The qRT-PCR was carried out on the CFX96^TM^ Real-Time System (C1000^TM^ Thermal Cycler, Bio-Rad) according to the manufacturer instructions. All the qRT-PCR reactions were performed in the 10 μL total sample volume including 5 μL SYBR Premix Go Taq (Promega, China), 0.25 μL each primer (10 mM), 3 μL nuclease-free water and 1.5 μL diluted cDNA. The reaction conditions were carried out as follows: 95 °C for 5 min, followed by 41 cycles of 95 °C for 15 s and 60 °C for 35 s. After the qRT-PCR cycles, Melt curve analysis of each qRT-PCR sample was performed to confirm each primer specificity: 95 °C for 1 min followed by a constant increase (0.25 °C per 1 s) of the temperature between 65 °C and 95 °C. The results of melt curve analysiss showed that only one product of each gene existed. The NTC (no template control) and NRT (no reverse transcription control) experiments were also carried out for each gene analysis. Tomato *SlCAC*^91^ was used as the internal standards. The 2^−∆∆C^T method^92^ was used to analyze the relative quantification of specific mRNA levels. The expression levels of ethylene synthesis and response genes, ripening-related genes and carotenoid synthesis genes were detected in fruits. All the primers of related genes used for qRT-PCR analysis are listed in Supplementary Table S2.

### Pigments content determination

For the total carotenoid extraction, the sample (1 g) of each line was cut from the same area of pericarp around the equator of fruits at B, B + 4 and B + 7 stage. After being triturated in the liquid nitrogen, 10 mL hexane:acetone (60:40, v/v) was used and then the total carotenoids of each sample was extracted. Then the extract was centrifuged for 5 min at 4000 g, the absorbance of the samples was immediately examined at 450 nm. The carotenoid content of each samples was calculated as follows: total carotenoid (mg/mL) = 4 × (absorbancy at 450 nm) × 10 mL/1 g^93,94^.

The lycopene extraction was performed according to the method described by Fish, W. W. *et al*.^95^. 0.4 to 0.6 g sample of each line was cut from the same area of pericarp around the equator of fruits at B, B + 4 and B + 7 stage. The 50 mL centrifuge tubes were used in this experiment. Three independent experiments of each sample were carried out. After being triturated in the liquid nitrogen, 20 mL 0.05% (w/v) BHT in acetone: 95% ethanol: hexane (1:1:2, v/v) was used. Then the 50 mL centrifuge tubes were laid on a container that contained ice and shaked in a orbital shaker to mix for 15 min at 180 rpm. After shaking was finished, the ice deionized water (3 mL) were added into each tube, and then the samples were shaken to mix for another 5 min at 180 rpm. After 5 min of shaking, the tubes were left for 5 min at room temperature to allow for phase separation. The supernatant (hexane layer) was used to measure the absorbance at 503 nm. The quartz cuvette was 1 cm path length and the hexane solvent was used as the blank control. The carotenoid content of each samples was calculated as follows: Lycopene (mg/kg) = (A_503_ × 31.2)/g tissue^95^.

### Ethylene measurements

The B, B + 4, B + 7 fruits of transgenic lines and the wild type tomato were used and put in a open jars (100 mL) for 3 h to minimize the impact of ethylene that induced by the wound of fruits picking. After being sealed, the jars were stored at the room temperate for 24 h. Then 1 mL of the headspace gas of each sample was injected into the gas chromatograph (Hewlett-Packard 5890 series) using the flame ionization detector. Each sample was normalized for fruit weight compared to the ethylene standards which concentration had been known^81^. Three independent experiments were performed for each sample.

### Yeast two-hybrid assay

The MATCHMAKER GAL4 Two-Hybrid System III was used to perform the yeast two-hybrid assay following the method described by the manufacturer (Clontech). The PCR experiments were performed to amplify the open reading frame (ORF) of *SlCMB1* using the primers *SlCMB1*(Y2H)F/R (Supplementary Table S1). The products were cloned into the *Eco*R I/*Bam*H I site of the pGADT7 vector to generate the pGADT7-*SlCMB1* vector (Supplementary Fig. S4B). Meanwhile, the ORFs of *SlMADS-RIN*, *SlMADS1*, *SlAP2a* and *TAGL1* were also amplified using the primers *SlMADS-RIN*(Y2H)-F/R, *SlMADS1*(Y2H)-F/R, *SlAP2a*(Y2H)-F/R and *TAGL1*(Y2H)-F/R (Supplementary Table S1), respectively. After being digested by *Bam*HI and *Eco*RI, the products were linked into the *Eco*RI/*Bam*HI site of the vector pGBKT7 to generate the pGBKT7-*RIN*, pGBKT7-*SlMADS1*, pGBKT7-*SlAP2a* and pGBKT7-TAGL1 (Supplementary Fig. S4A), respectively. The pGADT7-*SlCMB1* vector was transferred into Y187 and the pGBKT7-*RIN*, pGBKT7-*SlMADS1*, pGBKT7-*SlAP2a* and pGBKT7-*TAGL1* were also transferred into Y2Hgold, respectively. The Y187 with prey (pGADT7-*SlCMB1*) was plated on SD (synthetic dropout) medium without Leu. The Y2HGold with the pGBKT7-*RIN*, pGBKT7-*SlMADS1*, pGBKT7-*SlAP2a* and pGBKT7-*TAGL1* bait was plated on the SD medium without Trp (SDO), respectively. In parallel, the self-activation experiments of pGBKT7-*RIN*, pGBKT7-*SlMADS1*, pGBKT7-*SlAP2a* and pGBKT7-*TAGL1* were performed on the SD medium with no Trp, His and adenine (TDO) (Supplementary Fig. S5), respectively. Then, the Y2HGold with bait (pGBKT7-*RIN*, pGBKT7-*SlMADS1*, pGBKT7-*SlAP2a* and pGBKT7-*TAGL1*) and the Y187 with prey (pGADT7-*SlCMB1*) were cultured together in the 2 × YPDA medium at 200 rpm for 24 h, respectively. The cultures were plated on the SD medium with no Leu and Trp (DDO) to select the diploids containing the prey and bait vectors, simultaneously. 3 to 5 days later, the fresh diploid cells were cultured on the SD medium which lacked adenine, His, Leu and Trp, with the indicator X-a-Gal (QDO/X) to confirm whether SlCMB1 could interact with SlMADS-RIN, SlMADS1, SlAP2a and TAGL1 or not, respectively. All plates were incubated at 30 °C for 3–5 d. The empty bait and prey vector containing the construct of each bait and prey were used as negative controls, respectively. In parallel, positive controls were also cultured. Three independent repetition of these experiments were performed with fresh transformants.

### Statistical analysis

The mean values of data were measured from three replicates and ‘Standard Error’ of the means was calculated. Data were analyzed by Origin 8.0 software, and t test (SAS 9.2) was used for assessing significant differences among the means.

## Electronic supplementary material

Supplementary information
