# Supplementary material for: A tomato MADS-box protein, SlCMB1, regulates ethylene biosynthesis and carotenoid accumulation during fruit ripening
Source: Sci Rep. 2018 Feb 21;8:3413. doi: 10.1038/s41598-018-21672-8 (PMC5821886; doi:10.1038/s41598-018-21672-8)
Supplement: Supplementary file 1 — Supplementary information [file 41598_2018_21672_MOESM1_ESM.pdf]

Supplementary information

A tomato MADS-box protein, SlCMB1, regulates ethylene biosynthesis and carotenoid accumulation during fruit ripening

Jianling Zhang, Zongli Hu, Qiyuan Yao, Xuhu Guo, Vanluc Nguyen, Fenfen Li Guoping Chen *

Laboratory of molecular biology of tomato, Bioengineering College, Chongqing University, Chongqing, People's Republic of China

* **Corresponding author:** Guoping Chen

Bioengineering College, Chongqing University, Chongqing, People's Republic of China

Phone: 00862365112674

Fax: 00862365112674

E-mail: chenguoping@cqu.edu.cn

Supplementary Table S1. Specific primer sequences used for *SlCMB1* gene amplification and cloning procedures.

Table S1. Details of primers for qPCR amplification

Primer Name	Primer Sequence (5'–3')	Application
SlCMB1-i	CGGGGTACCAAGCTTACTCTGTAGCACATTGGCATATCAC<br>CCGCTCGAGTCTAGAAGATTAGTATGAATATCACGCACAAGTC	To establish *SlCMB1* RNAi lines; add *Kpn*I+ *Hind* III and *Xho*I +*Xba*I site underlined, respectively
SlCMB1-Full	TACATAGCCTTTTTTCTTTTCCT<br>TGAATATCACGCACAAGTCG	Full-length amplification of Positive transgenic plants
NPT II	CTCAGAAGAACTCGTCAAGAAGG<br>GACTGGGCACAACAGACAATC	
SlCMB1(Y2H)	CCGGAATTCATGGGAAGAGGTAAGGTAGAATTGA<br>CGCGGATCCTCAGATTCTGAATTCGCCCCT	Construction of yeast two -hybrid vector
RIN(Y2H)	CCGGAATTCATGGGTAGAGGGAAAGTAGA<br>CGCGGATCCTCATAGATGTTTATTCAT<br>CGCGGATCCTTAAAGCATCCATCCATGAATA	
SlMADS1(Y2H)	CCGGAATTCATGGGAAGAGGAAGAGTTG<br>CGCGGATCCTTAAAGCATCCATCCATGAATA	
AP2a(Y2H)	CCGGAATTCATGTGGAATTTAAATGATTCCCC<br>CGCGGATCCTCAAGGTCTCATAAAATAATGATGGA	
TAGL1(Y2H)	CCGGAATTCATGGTTTTTCCTATTAATCAGG<br>CGCGGATCCTCAGACAAGCTGGAGAGGAG	

Supplementary Table S2. Specific primer sequences used for qRT-PCR analysis

Table S2. Details of primers for qPCR amplification

Primer Name	Primer Sequence (5'–3')	Product (bp)
SlCAC	CCTCCGTTGTGATGTAACTGG<br>ATTGGTGGAAAGTAACATCATCG	173 bp
q-SlCMB1	TGAGCGTCAACTGGATTCATCTT<br>CCCTCTGACTGAGCAGGTTGTT	213 bp
ACO1	ACAAACAGACGGGACACGAA<br>CTCTTTGGCTTGAAACTTGA	181 bp
ACO3	CAAGCAAGTTTATCCGAAAT<br>CATTAGCTTCCATAGCCTTC	113 bp
ACS2	GAAAGAGTTGTTATGGCTGGTG<br>GCTGGGTAGTATGGTGAAGGT	107 bp
ACS4	GCTCGGAGGTAGGATGGTTTC<br>GTTCCTCTTCCATTGTGCTTGT	151 bp
E4	AGGGTAACAACAGCAGTAGCA<br>CCCAACCTCCGTCTTCAC	167 bp
E8	GGCACCATTCAACATACCG<br>CTTTCACCGAAGAAGCACG	242 bp
ERF1	TTTTAGTATCGGATGGACG<br>GGCGGAGAAACAGAAGTA	102 bp
PSY1	AGAGGTGGTGGAAAGCAA<br>TCTCGGGAGTCATTAGCAT	298 bp
PDS	GCTTTACCCGCTCCTTTA<br>ACCTTGCTTTCTCATCCA	174 bp
LCYB	TTGACTTAGAACCTCGTTATTGG<br>AACAGTTCCCTTTGTCATTATCTC	137 bp
LCYE	GCCACAGGTTATTCAGTCGTCA<br>CCAGTCCAAATAGGAAAAACGAT	196 bp
CYCB	CGACGTGATCATTATCGGAGC<br>GTGGTGAAGGGTCAACACAACA	98 bp
RIN	CATCATGGCATTGTGGTGAGC<br>AATTCAAAGCATCCATCCAGGTAC	194 bp
TAGL1	CGCAATAACTCCCTGCCTGTA<br>GAAGATGAAGAGCCTTGACCC	143 bp
FUL1	AAAATCAGTGGGAAATCAACTCATC<br>CCTTGCTGCTGTGAAGAACTACC	139 bp
FUL2	CCGTGGGAGCAACAGAGTCAT<br>GGAGGCATCACAGAAGCACTG	167 bp
LoxC	TTTACTCCGCCCTACACGC<br>CCTGAAAGATCGACACCCA	121 bp
PE	GCTTGCGTCTTTGACAACTCAGG<br>GTGCCACCACTGCATTCGCTAT	137 bp

Supplementary Figures

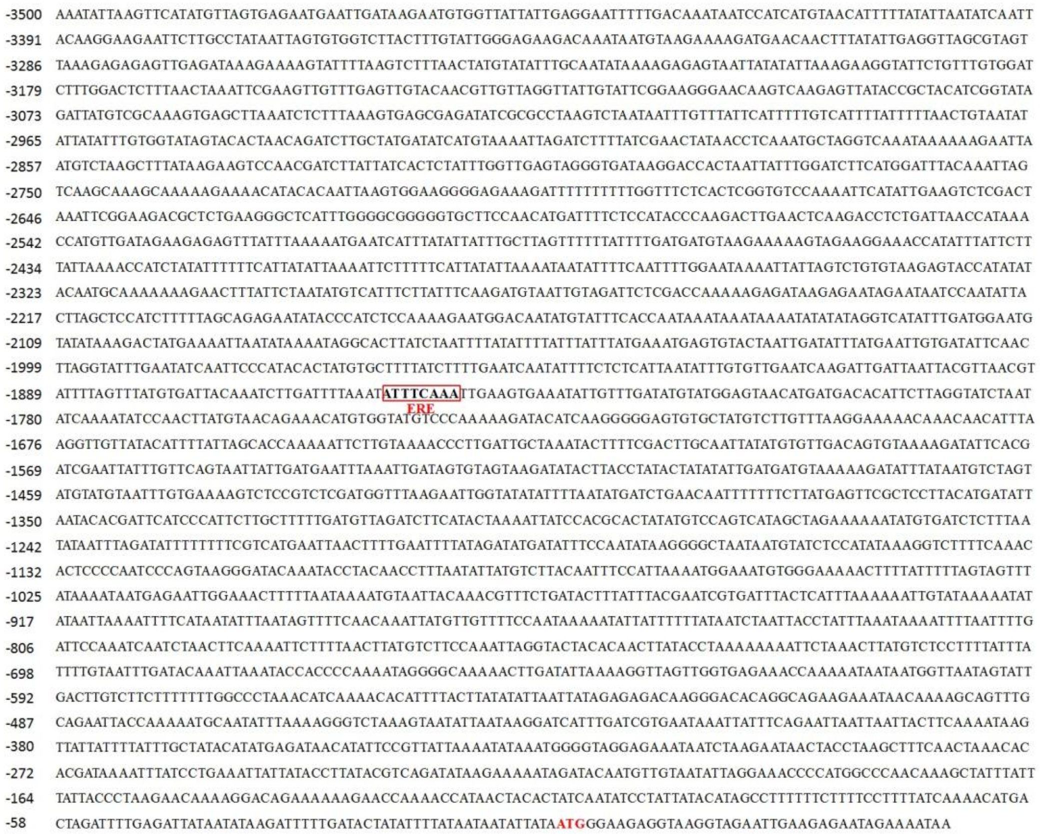

Supplementary Fig. S1. Promoter analysis of *SlCMB1* gene. Promoter sequence (3500 bp regions upstream the 5' end of the predicted ORF) of *SlCMB1* gene was extracted from SGN database and searched against the promoter database plant CARE (http://bioinformatics. Psb.ugent.be/webtools/plantcare/html/), ERE motif (ATTTCAAA) are the ethylene-responsive element in *SlCMB1* promoter region.

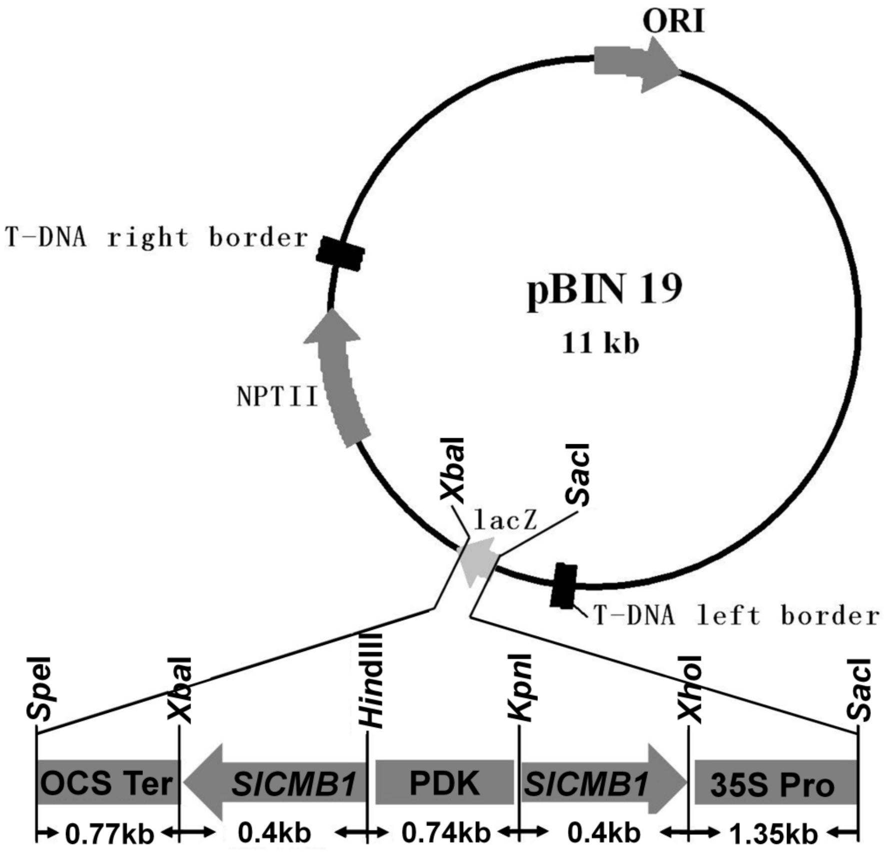

Supplementary Fig. S2. Hairpin construct of the *SlCMB1* gene for double-stranded RNAi vector. The *SlCMB1* gene-specific sequence in the antisense and sense orientations were linked with a PDK gene fragment and as a transcriptional unit for hairpin RNA expression which premoted by the CaMV 35S promoter and terminated by the OCS terminator. Among which, *Spe*I and *Xba*I are isocaudamers.

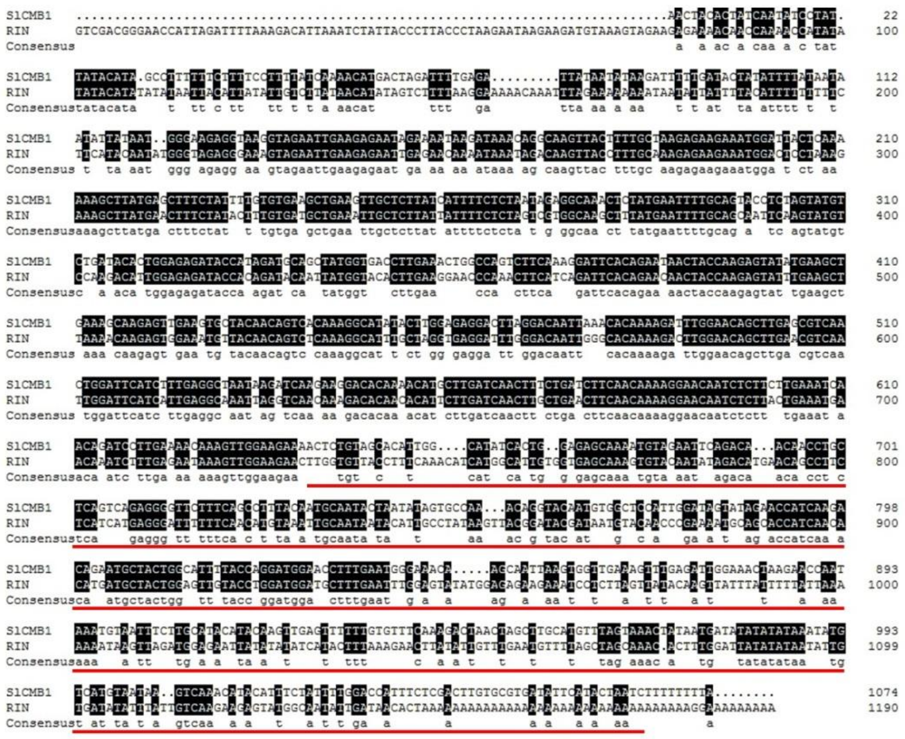

Supplementary Fig. S3. Multiple sequence alignment of *SlCMB1* and *RIN*. The 426 bp 3' specific fragment of *SlCMB1* used in this study is indicated by the red line.

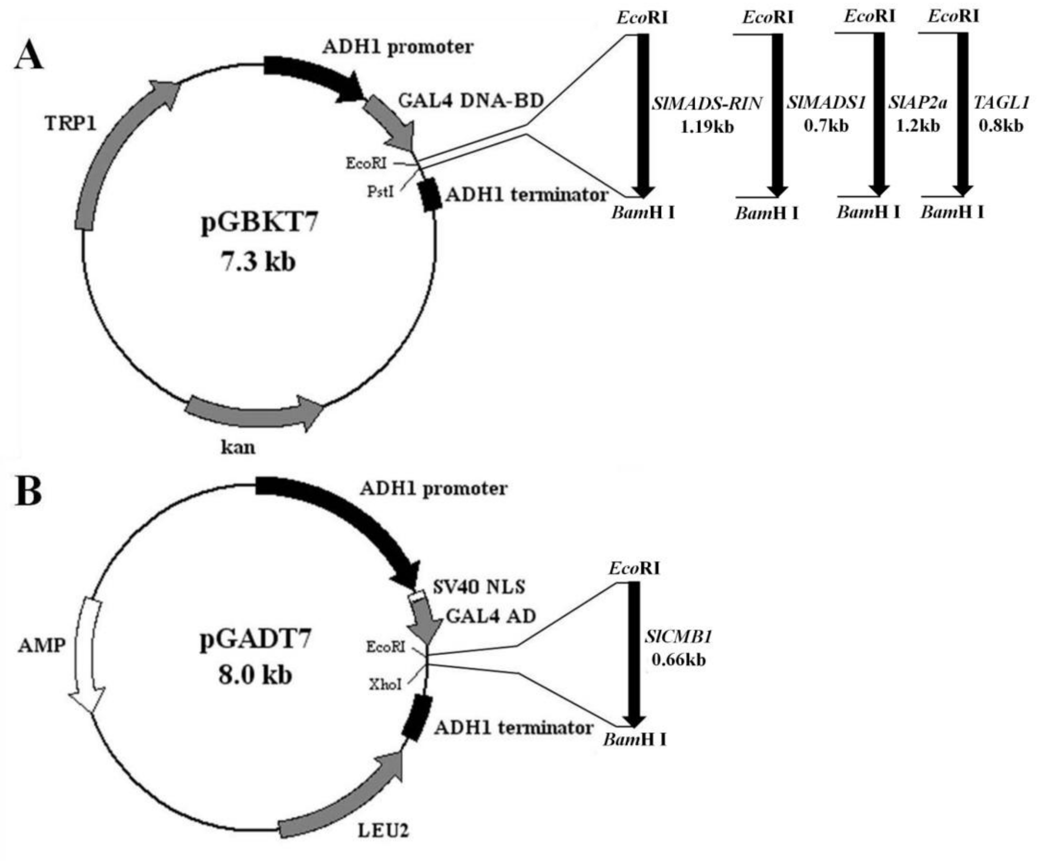

Supplementary Fig. S4. Construct of SlCMB1 and RIN gene for yeast two-hybrid vector. (A) The ORFs of *SlMADS-RIN*, *SlMADS1*, *SlAP2a*, *TAGL1* were cloned into pGBKT7 bait vector to obtain the vector pGBKT7-*SlMADS-RIN*, pGBKT7-*SlMADS1*, pGBKT7-*AP2a* and pGBKT7-*TAGL1*, respectively. (B) The ORF of *SlCMB1* was cloned into pGADT7 prey vector to obtain the vector pGADT7-*SlCMB1*.

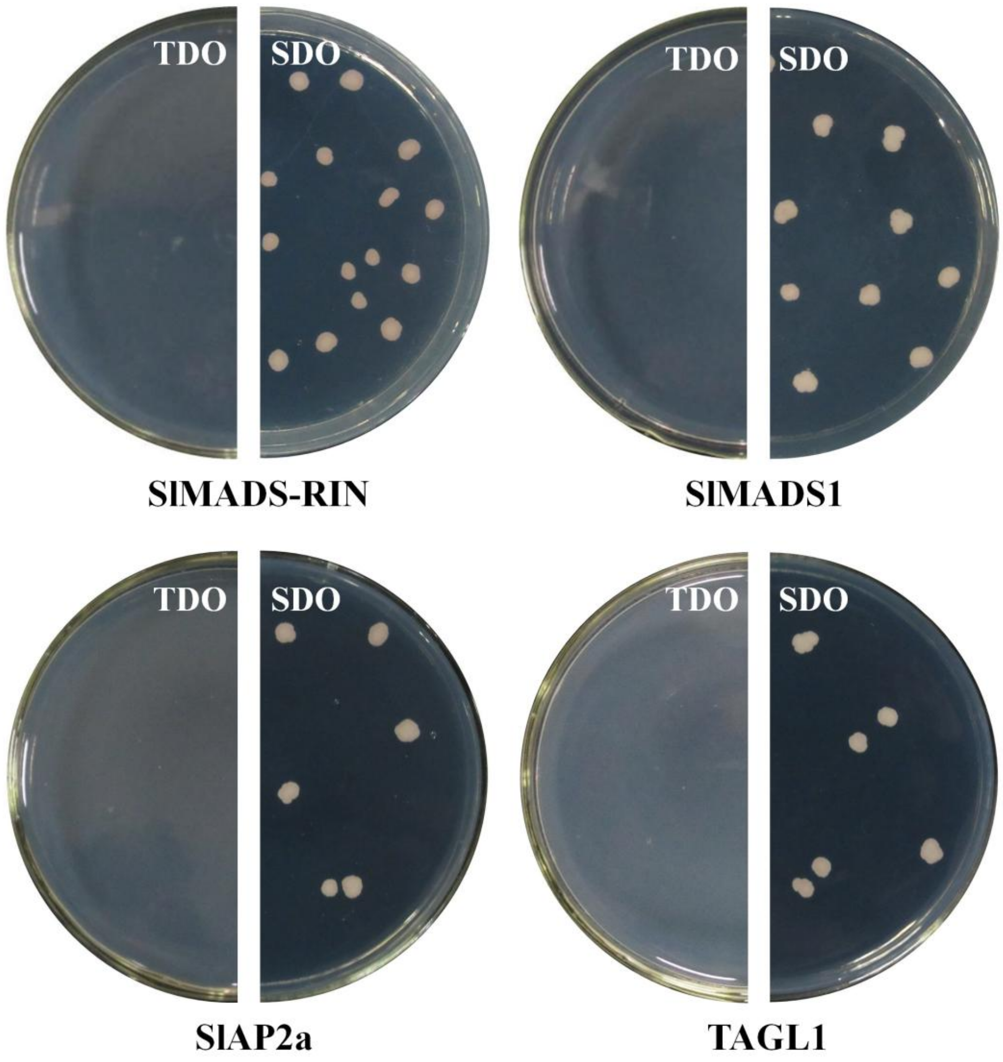

Supplementary Fig. S5. Yeast two-hybrid assay for SlCMB1 and SlMADS-RIN, SlMADS1, SlAP2a and TAGL1. TDO, SD medium without Trp, His, Ade (autoactivation assay); SDO, SD medium without Trp (autoactivation assay).
